# Behaviors toward Noncommunicable Diseases Prevention and Their Relationship with Physical Health Status among Community-dwelling, Middle-aged and Older Women in Indonesia

**DOI:** 10.3390/ijerph17072332

**Published:** 2020-03-30

**Authors:** Masako Yamada, Elsi Dwi Hapsari, Hiroya Matsuo

**Affiliations:** 1 Graduate School of Health sciences, Kobe University, Kobe 654-0142, Japan; buffalogrove0816@gmail.com; 2 Department of Pediatric and Maternity Nursing, Faculty of Medicine, Public Health, and Nursing, Universitas Gadjah Mada, Yogyakarta 55281, Indonesia; elsidh@ugm.ac.id

**Keywords:** noncommunicable disease, prevention, behavior, community health, health promotion

## Abstract

We aim to clarify the behaviors toward noncommunicable diseases (NCDs) prevention focusing on lifestyle-related diseases and physical health status and examine their relationship among community-dwelling women in Indonesia. This cross-sectional study included women aged 45 years and older. Data were collected through an interview using a structured questionnaire; the following parameters were also measured: height, weight, body mass index (BMI), blood pressure (BP), handgrip strength, and 10 m gait speed. This study found that the majority of women adopted one or more healthy behaviors to prevent NCDs, while few women practiced comprehensive behaviors. Age, satisfaction with house income, living alone, social support, social participation, and household decision making were the determinant factors for behaviors toward NCDs prevention. A high prevalence of underweight (26.4%), overweight (31.9%), obesity (5.6%), high systolic blood pressure (SBP) (62.5%), and low muscle strength (54.2%) were frequently observed. Eating well-balanced meals, avoiding fatty foods, and undergoing blood cholesterol testing had significant correlations with physical health status. It is concluded that the activities at Posyandu Lansia (health village posts for older adults) are necessary to help women with unhealthy eating behaviors and lower physical activity and unawareness of health checkups to maintain focus and to develop a more practical approach to NCDs prevention.

## 1. Introduction

According to the World Health Organization (WHO), noncommunicable diseases (NCDs), such as cardiovascular diseases, cancers, chronic respiratory diseases, and diabetes, account for 41 million deaths each year globally, and more than three-quarters of global NCD-related deaths occur in low- and middle-income countries [1]. Indonesia is one of the countries that has been facing a rapid increase in NCD-related deaths [2,3], and the prevalence of NCDs including obesity, hypertension, and diabetes in women is significantly higher than that in men [4,5,6,7,8]. The Indonesian Ministry of Health has developed NCDs control programs and launched several community health centers (Puskesmas) and health village posts for older adults (Posyandu Lansia) in the mid-2000s. Current activities at Posyandu Lansia are mostly focused on disease and risk factor screening, such as blood pressure (BP) or body weight measurement, while a few also implement activities related to disease prevention, health promotion, and social engagement [9]. Many studies have reported the rapid increase of NCDs, but there has been only a little research on how the community may be involved in the successful management of NCDs [10]. A previous study reported that the participants with hypertension living in rural Indonesia only adopted healthy-lifestyle behaviors for personal reasons, such as behavioral beliefs and cultural competence. These personal reasons were reinforced by social reasons, such as religious support, social support, and health system support [11]. Moreover, women living in rural areas did not have enough time to perform exercise as they were busy working both at home and in the fields [12]. It is expected that individuals living healthily not only survive longer but live longer in better health, with the occurrence of disability and age-related diseases postponed to the last years of life [13]. For this reason, the prevention and control of NCDs and deterioration of activities of daily living (ADL) and quality of life (QOL) have become a major challenge particularly for middle-aged and older women because women have greater roles in family and community health activities in Indonesia [14]. However, no study has yet demonstrated the behaviors toward NCDs prevention and their relationship with physical health status among community-dwelling, middle-aged and older women in Indonesia. Therefore, this study aims to clarify the behaviors adopted to prevent NCDs focusing on lifestyle-related diseases and physical health status and examine the associations between behaviors adopted to prevent NCDs and physical health status among community-dwelling, middle-aged and older women in Indonesia.

## 2. Materials and Methods

### 2.1. Participants

This cross-sectional study included community-dwelling women aged 45 years and above who participated in monthly activities for Posyandu Lansia from March to June 2019. A total of 72 women were recruited in this study. Eight Posyandu lansia locations were selected from the three subdistricts (Godean, Mlati, and Pakem) of Sleman district, special region of Yogyakarta province. Women who had activities of daily living (ADL) disabilities assessed using the Barthel Index and who used electronic medical devices including a pacemaker were excluded.

### 2.2. Methods

#### 2.2.1. Questionnaires

Data were collected via face-to-face interview using structured questionnaires. The questionnaire was developed based on the preliminary study conducted in October 2018 among 24 health cadres living in one rural area in Yogyakarta and in collaboration with the Indonesian research partners. The questionnaires had five components: participant’s characteristics, lifestyle, understanding of NCDs terms, social relationships, and behaviors toward NCDs prevention. Participant’s characteristics consisted of age, duration of residence, marital status, living arrangement, education, satisfaction with house income, and past history. Lifestyle was assessed using the FANTASTIC lifestyle assessment scale developed by Wilson et al. [15]. This instrument was composed of 25 items, with scores ranging from 0 to 50. Items related to lifestyle were rated as follows: in control (42–50), on the right track (35–41), fair (30–34), somewhat low (20–29), and in the danger zone (0–19). Three of the items in the scale were used to assess the participant’s understanding of NCDs terms, such as lifestyle-related disease, menopause, and sarcopenia. Another three items were used to assess social relationships, such as social participation, social support, and household decision making. The social participation questionnaire developed by Utomo et al. [14] comprised 10 questions, but the instrument was modified into a 9-item questionnaire, which was suitable to the participants of this study. Participants whose scores (7–9) were higher than the average score were categorized as higher group, while participants whose scores (0–6) were lower than the average score were categorized as the lower group. Social support was assessed using the social network scale developed by Lubben et al. [16]. The scale is composed of 6 items that assess social isolation, with scores ranging from 0 to 30. The scores were divided into two levels: isolated (0–12) and not isolated (13–30). The household decision-making scale, which includes three items, was developed by Leon et al. [17]. Responses were rated as follows: by myself (1), spouse/partner (2), spouse/partner jointly (3), and someone else (4). The behaviors toward NCDs prevention scale consisted of 11 items, including eating behavior, physical activity, sleep, and health checkups based on Breslow’s healthy habits and question items used by previous related studies.

#### 2.2.2. Physical Measurements

Height was measured using a roll ruler wall mounted height stadiometer, which was placed at each Posyandu Lansia. Weight was measured, with the participant not wearing shoes and socks, using a weight scale (body scan, HBF-702T; Omron Healthcare Co. Ltd., Kyoto, Japan). Body mass index (BMI) was defined as the weight in kilograms divided by the square of the height in meters (kg/m^2^) and classified by age group (middle-aged women and older women) [11]: underweight (<20.0 kg/m^2^), normal weight (20.0 to <25.0 kg/m^2^), overweight (25.0 to <30.0 kg/m^2^), and obesity (≥30.0 kg/m^2^). Two consecutive measurements of systolic BP (SBP) and diastolic BP (DBP) were recorded using a blood pressure monitor (HEM-7080-IC; OMLON CO.,Ltd., Kyoto, Japan). The arithmetic average of the two BP measurements was calculated. Hypertension was defined as SBP ≥140 mmHg and/or DBP ≥90 mmHg according to the WHO criteria (https://www.who.int/news-room/fact-sheets/detail/hypertension). Handgrip strength was measured using an analog hand dynamometer (T.K.K. 5001; Takei Scientific Instruments Co., Ltd., Niigata, Japan) twice on each side; the greater value was used for analysis. For the 10 m walk test, two end lines and two buffer lines were taped on the ground. Each end line measured 16 m from the other line, while each buffer line measured 3 m from the end line. The buffer lines were used to control acceleration and deceleration. The time it took to walk the middle 10 m was recorded using a stopwatch. Participants were instructed to walk twice at their self-perceived comfortable pace; the less value was used for analysis.

### 2.3. Data Analyses

All statistical analyses were conducted using IBM SPSS Statistics ver. 24.0 (IBM CO., Ltd., New York, USA) and EZR (Saitama Medical Center, Jichi Medical University, Saitama, Japan), which is a graphical user interface for R (The R Foundation for Statistical Computing, Vienna, Austria). Descriptive statistics were used to describe the variables. The bivariate analysis for categorical variables was performed using the Fisher’s exact test to determine the associations between participants’ characteristics, understanding of NCDs terms, social participation, social isolation, and household decision making. Post hoc multiple comparison was performed using the Holm method. A multivariate analysis was performed with multiple regression analysis to examine the association between behaviors toward NCDs prevention and physical health status. In all cases, *p*-values of <0.05 were considered significant.

### 2.4. Ethical Consideration

Participants were informed about the study aim and methods and were assured about voluntary participation and privacy protection. A written informed consent was obtained from all participants. The study was approved by the Ethical Review Committee of Kobe University Graduate School of Health Sciences (No.813) and Gadja Mada University (3/0/UN1/FKKMK/KAM.2/LT/2019).

## 3. Results

### 3.1. Participants’ Characteristics

Table 1 shows the characteristics of the participants. The mean age was 63.4 ± 9.9 years (mean ± S D). Of the 72 participants, 33.3% (*n* = 24) were aged 45–59 years, while 66.7% (*n* = 48) were aged 60 years and above. Approximately 77.9% (*n* = 53) of the participants have lived in the current address for over 30 years. Majority of the participants (98.6%) were either married or widowed. Most of participants (75.0%) were living with their spouses or son, while 9.7% were living alone. Approximately 34.7% of the participants have never attended or not completed primary school, 30.6% had primary education, and 34.7% had secondary education. 72.2% of the participants were satisfied with their house income to cover their daily needs. Approximately one quarter of the participants have never been diagnosed with hypertension, diabetes, and/or hyperlipidemia by a doctor.

### 3.2. Lifestyle, Understanding of NCDs Terms, and Social Relationships

Table 2 shows the lifestyle, understanding of NCDs terms, and social relationships of the participants. Questions related to lifestyle were rated as follows: in control (8.3%), on the right track (62.5%), fair (22.2%), somewhat low (6.9%), and in the danger zone (0.0%). The proportions of participants who understood the terms lifestyle diseases, menopause, and sarcopenia were 41.7%, 26.4%, and 1.4%, respectively. Approximately 72.2% of the participants participated in seven or more social activities. Meanwhile, 15.3% demonstrated social isolation according to the Lubben Social Network Scale [16]. The percentages of participants who usually made household decisions by themselves were as follows: major household purchases 38.9%, purchases for household daily needs 43.1%, and visits to family or relatives 36.1%.

### 3.3. Behaviors toward NCDs Prevention

Table 3 shows the behaviors toward NCDs prevention. Most of the participants (80.6%) practiced one or more behaviors toward NCDs prevention. The proportions of participants who practiced NCDs prevention were as follows: avoided salty foods, 55.6%; consumed a well-balanced diet, 50.0%; slept well, 47.2%; avoided foods with high sugar content, 43.1%; performed regular exercise, 43.1%; ate breakfast daily, 40.3%; taking a walk, 34.7%; and avoided fatty foods, 23.6%. Approximately 87.5% of the participants had their BP checked in the past month, 55.6% underwent blood sugar testing, and 38.9% underwent blood cholesterol testing within the past year. The reasons for not undergoing blood sugar testing or blood cholesterol testing were as follows: “I do not feel the necessity because I am not sick” (50.0%), “I need to go to see a doctor if I get sick” (9.5%), and “It costs too much” (9.5%).

### 3.4. Physical Measurements

Table 4 shows the results of the physical assessment. The mean BMI was 23.2 ± 4.3. The participants’ BMI values were categorized as follows: underweight (<20.0 kg/m^2^, 26.4%), normal weight (20.0 to <25.0 kg/m^2^, 33.3%), overweight (25.0 to <30.0 kg/m^2^, 31.9%), and obesity (≥30.0 kg/m^2^, 5.6%). The mean SBP was 151.4 ± 26.3, while the mean DBP was 84.3 ± 13.8. The participants’ SBP and DBP values were categorized as follows: normal SBP (SBP <140 mmHg, 37.5%), high SBP (140 mmHg ≤ SBP, 62.5%), normal DBP (DBP <90 mmHg, 72.2%), high DBP (90 mmHg ≤ DBP, 27.8%). The mean handgrip strength was 15.7 ± 5.0 kg, and the mean gait speed was 1.0 ± 0.2m/s. The percentages of participants with <18 kg in handgrip strength and <0.8m/s gait speed, which was the cutoff value of sarcopenia, were 54.2% and 11.1%.

### 3.5. Factors associated with behaviors toward NCDs prevention

Table 5 and Table 6 show the factors associated with behaviors toward NCDs prevention. Participants who were satisfied with their house income were more likely to eat breakfast daily than those who were not satisfied with their household income (*p* = 0.037). Participants who had been residing at their current address for 30 years and more tended to intake less sugar than those who had been residing for 20–29 years (*p* = 0.017). In addition, participants who participated in seven or more social activities (higher group) tended to intake less sugar than participants who participated in less than seven social activities (lower group) (*p* = 0.010). Participants who were not living alone tended to intake less salt than those who were living alone (*p* = 0.013). Participants who did not demonstrate social isolation tended to intake less salt than those who demonstrated social isolation (*p* = 0.035). Participants who were aged 60 years old and older tended to take a walk more than those aged 45–59 years old (*p* = 0.016). Participants who made household decisions (purchases for daily needs) by themselves tended to undergo BP monitoring more regularly than those who made household decisions by their husbands (*p* = 0.002). 

### 3.6. Association between Behaviors toward NCDs Prevention and Physical Health Status

Table 7 shows the association between behaviors toward NCDs prevention and physical health status. The multiple regression analysis revealed that avoiding fatty foods was significantly correlated with SBP (*p* = 0.028), while well-balanced meals and blood cholesterol testing were significantly correlated with hand grip strength (*p* = 0.018, *p* = 0.009).

## 4. Discussion

This study found that the majority of community-dwelling, middle-aged and older women adopted one or more healthy behaviors to prevent NCDs, while few women practiced healthy behaviors comprehensively, including eating behavior, physical activity, sleep, and health checkups. Moreover, only a few were motivated to avoid fatty foods and take a walk. In addition, a high prevalence of overweight/obesity and high BP, underweight, and muscle weakness were frequently observed in this study. It was also indicated for the first time that eating behavior and health checkup behavior have a correlation with physical health status.

First, many of the women paid some attention to their health and practiced healthy behaviors to prevent NCDs, while women who adopted healthy behaviors comprehensively, such as eating behavior, physical activity, sleep, and health checkups, were limited in this study. In addition, the proportions of participants who avoided fatty foods and were encouraged to walk were lower than those who practiced other behaviors. The 2013 National Basic Health Survey also reported the proportion of people who tended to consume fatty foods was high (40.7%); it was further indicated that women tended to consume excessive fat compared to men [18]. Many Indonesians are fond of consuming fried foods or coconut oil; these foods are rich in saturated fatty acids and promote atherosclerosis by excessive intake [19]. Therefore, failure to avoid fatty foods and perform physical exercise like walking could increase the risk of atherosclerosis among middle-aged and older women. Moreover, excessive intake of salt and sugar could increase the risk atherosclerosis, thus leading to the development of cardiovascular diseases [20,21]. This study also found that living alone, socially isolated, and socially inactive were factors associated with increased intake of salt and sugar. Therefore, it was indicated that middle-aged and older women who were living alone, in social isolation, and with a low level of social participation were more likely to have unhealthy eating behaviors. In addition, younger generations aged below 60 years were less likely to walk than those aged over 60 years. Exercises that increase oxygen intake such as walking are effective in improving BP, blood sugar levels, and blood cholesterol levels by eliminating fat and by reducing glycated hemoglobin (A1c) and improving metabolism [22]. However, women in their 40s and 50s were busy performing housework, childcare, and work in the field or farm; hence, it is difficult for them to continue and maintain exercise habits in their leisure time. Moreover, Indonesia has a tropical climate; therefore, it is not easy to perform physical activities as the temperature is high throughout the year. Thus, it is necessary to include activities for Posyandu Lansia that can increase the amount of physical activity without experiencing difficulties, which are suitable for their living style and individual physical strength.

With regard to physical measurement, the proportion of underweight, overweight, and obese participants were high. The proportion of women with high BP in this study is the same as that reported in the 2013 National Basic Health Survey. However, the mean handgrip strength reported in this research was lower than that of a previous study [23]. Obesity was associated with not only chronic diseases, such as hypertension, diabetes, and dyslipidemia, but also with a decline in ADL, frailty, and motor dysfunction at older age [24,25]. Moreover, being underweight due to poor nutritional status may rapidly cause a decrease in muscle strength or osteoporosis [26]. In addition, the age-related decline in muscle mass and strength could cause the decline in ADL and QOL [27]. Therefore, middle-aged and older women may be at a higher risk of age-related health problems, such as a decrease in muscle mass and strength (sarcopenia) and weakness as well as chronic diseases, such as obesity/overweight, underweight, and high BP.

Finally, eating behaviors and health checkup behaviors were correlated with physical health status, such as BP and handgrip strength. Eating behaviors such as avoiding fatty foods and consuming a well-balanced diet caused a decrease in BP and increase in handgrip strength. Fried food and foods rich in animal fat have been found to increase atherosclerosis and further elevate BP (Hooper et al. 2001). Inappropriate diet and nutrition have also been associated with the decline in physical function and muscle function [28]. These findings highlight the possibility that women who have appropriate eating behaviors, such as avoiding fatty foods and eating well-balanced meals, could pay attention to their health in general and also have more chances to be physically active. Second, health checkup behaviors such as undergoing a blood cholesterol testing was associated with handgrip strength. As blood cholesterol testing was considered optional and was not free of charge in this research area, only those who underwent this testing were more likely to be aware of their health and more conscious of their health status. Therefore, women who were more aware of their health status and practice behaviors that prevent NCDs were more likely to have muscle strength.

The activities for Posyandu Lansia will be further enhanced [29] so that middle-aged and older women in Indonesia can maintain and promote appropriate health behaviors to prevent NCDs. Opportunities that can allow these women to determine their unhealthy habits, recognize the necessity of lifestyle modification, and learn more practical programs, such as physical exercise and health education for nutrition, are needed. Especially, it is important to support women who are younger, living alone, socially inactive, socially isolated, or do not make household decisions by themselves to modify their unhealthy behaviors. In addition, it is essential to encourage middle-aged and older women to undergo health checkups, be aware of their own health status, and maintain their behaviors toward NCDs prevention. In order to improve their ability to maintain their health checkup behaviors, their husbands or their family member will serve as key factors as the family plays an important role in making decisions regarding the management of older people [30]. This study indicates that household decision making is related to having health checkups. Our findings supported the importance of increasing their awareness including their family members in order to accelerate women’s behaviors toward NCDs prevention.

Several limitations are considered in the present study. First, this study used cross-sectional design, which may have resulted in a limited result that cannot determine the causal relationship between behaviors toward NCDs prevention and physical health status. Therefore, a longitudinal study is needed to determine the causal relationship. Secondly, the small size of the sample in the present study might have affected the large standard error for the sample, which lowered the statistical power. From this view, further study is needed to examine the relationship between behaviors toward NCDs prevention and physical health status among middle-aged and older women in Indonesia. 

## 5. Conclusions

The findings indicated that middle-aged and older women in Indonesia practiced one or more healthy behaviors to prevent NCDs, while few women practiced comprehensive behaviors, including eating behavior, physical activity, sleep, and health checkups. Only a limited number of women avoided fatty foods and performed physical exercises like walking. Women who were living alone, socially inactive, and socially isolated had unhealthy eating behaviors. Women aged 60 years and older were more likely to be physically active. Women who made household decision by themselves were more likely to undergo health checkups. In addition, the high prevalence of underweight (26.4%), overweight (31.9%), obesity (5.6%), high SBP (62.5%), and low muscle strength (54.2%) cases were frequently observed in this study. Moreover, eating well-balanced meals, avoiding fatty foods, and undergoing blood cholesterol testing had a significant correlation with physical health status (*p* < 0.05). The activities at Posyandu Lansia are necessary to help women with limited eating behaviors and lower physical activity and unawareness of health checkups to maintain focus and to develop a more practical approach. It is also important to increase health awareness by actively involving women and their family.

## Figures and Tables

**Table 1:** Participants’ characteristics among middle-aged and older women in Indonesia.

Characteristics	*n*	(%)
**Age**		
45–59 years old	24	(33.3)
60 years and older	48	(66.7)
**Duration of residence**		
10–19 years	6	(8.8)
20–29 years	9	(13.2)
30 years and above	53	(77.9)
**Marital status**		
Married	59	(81.9)
Widow	12	(16.7)
Unmarried	1	(1.4)
**Living family**		
Living alone	7	(9.7)
Living with spouse or/and son	54	(75.0)
Other	11	(15.3)
**Education**		
Never attended/Not completed primary school	25	(34.7)
Primary school	22	(30.6)
Secondary school or higher	25	(34.7)
**Satisfaction with house income**		
Satisfied	52	(72.2)
Unsatisfied	11	(15.3)
**Past history**		
Yes	18	(25.0)
No	49	(68.1)

**Table 2:** Lifestyle, understanding of noncommunicable diseases (NCDs) terms, and social relationships among middle-aged and older women in Indonesia.

Lifestyle, Understanding of NCDs Terms and Social Relationships.		*n*	(%)
**Lifestyle**			
In control		6	(8.3)
On the right track		45	(62.5)
Fair		16	(22.2)
Somewhat low		5	(6.9)
In the danger zone		0	(0.0)
**Understanding of NCDs terms**			
Lifestyle-related diseases	No	42	(58.3)
	Yes	30	(41.7)
Menopause	No	53	(73.6)
	Yes	19	(26.4)
Sarcopenia	No	71	(98.6)
	Yes	1	(1.4)
**Social participation**			
Lower group		18	(25.0)
Higher group		52	(72.2)
**Social isolation**			
Not isolated		37	(51.4)
Isolated		11	(15.3)
**Household decision making**			
Major household purchases	By myself	28	(38.9)
	Spouse/partner	4	(5.6)
	Me and spouse/partner jointly	16	(22.2)
	Someone else	19	(26.4)
Purchases for household daily needs	By myself	31	(43.1)
	Spouse/partner	3	(4.2)
	Me and spouse/partner jointly	14	(19.4)
	Someone else	19	(26.4)
Visits to family or relatives	By myself	26	(36.1)
	Spouse/partner	4	(5.6)
	Me and spouse/partner jointly	20	(27.8)
	Someone else	17	(23.6)

**Table 3:** Behaviors toward NCDs prevention among middle-aged and older women in Indonesia.

Behaviors toward NCDs Prevention.		*n*	(%)
**Eating Behavior**			
Consume a well-balanced diet	Yes	36	(50.0)
	No	30	(41.7)
Eat breakfast daily	Yes	29	(40.3)
	No	37	(51.4)
Avoid foods with high sugar content	Yes	31	(43.1)
	No	35	(48.6)
Avoid salty foods	Yes	40	(55.6)
	No	26	(36.1)
Avoid fatty foods	Yes	17	(23.6)
	No	49	(68.1)
**Physical activity**			
Regular exercise	Yes	31	(43.1)
	No	35	(48.6)
Take a walk	Yes	25	(34.7)
	No	41	(56.9)
**Sleep**			
Sleep well	Yes	34	(47.2)
	No	32	(44.4)
**Health checkups**			
Blood pressure	Yes	63	(87.5)
	No	3	(4.2)
Blood sugar testing	Yes	40	(55.6)
	No	26	(36.1)
Blood cholesterol testing	Yes	28	(38.9)
	No	39	(54.2)

**Table 4:** Physical health status among middle-aged and older women in Indonesia.

Physical Health Status.	Mean ± SD	Classification	*n*	(%)
BMI	23.2 ± 4.3			
		<20.0	19	(26.4)
		20.0–24.9	24	(33.3)
		25.0–29.9	23	(31.9)
		30.0< =	4	(5.6)
Systolic blood pressure (mmHg)	151.4 ± 26.3			
		140–159	18	(25.0)
		160–180	15	(20.8)
		180< =	12	(16.7)
Diastolic blood pressure (mmHg)	84.3 ± 13.8	90< =	20	(27.8)
Hand grip strength (kg)	15.7 ± 5.0	<18	39	(54.2)
Gait speed (m/s)	1.0 ± 0.2	<0.8	8	(11.1)

**Table 5:** Factors associated with behaviors toward NCDs prevention among middle-aged and older women in Indonesia.

Factors	Eat Breakfast Daily					Avoid Foods with High Sugar Content					Avoid Salty Foods				
	No		Yes		*p*-Value	No		Yes		*p*-Value	No		Yes		*p*-Value
**Age**															
45–59 years old	14	(60.9)	9	(39.1)	0.611	15	(65.2)	8	(34.8)	0.198	10	(43.5)	13	(56.5)	0.792
60 years and older	23	(53.5)	20	(46.5)		20	(46.5)	23	(53.5)		16	(37.2)	27	(62.8)	
**Duration of residence**															
10–19 years	5	(83.3)	1	(16.7)	0.465	4	(66.7)	2	(33.3)	0.017 *	3	(50.0)	3	(50.0)	0.128
20–29 years	5	(55.6)	4	(44.4)		8	(88.9)	1	(11.1)		6	(66.7)	3	(33.3)	
30 years and above	25	(53.2)	22	(46.8)		19	(40.4)	28	(59.6)		15	(31.9)	32	(68.1)	
**Living alone**															
No	32	(54.2)	27	(45.8)	0.453	29	(49.2)	30	(50.8)	0.110	20	(33.9)	39	(66.1)	0.013 *
Yes	5	(71.4)	2	(28.6)		6	(85.7)	1	(14.3)		6	(85.7)	1	(14.3)	
**Satisfaction with income**															
Unsatisfied	10	(90.9)	1	(9.1)	0.037 *	8	(72.7)	3	(27.3)	0.314	6	(54.5)	5	(45.5)	0.498
Satisfied	25	(53.2)	22	(46.8)		24	(51.1)	23	(48.9)		18	(38.3)	29	(61.7)	
**Social participation**															
Lower	13	(76.5)	4	(23.5)	0.090	14	(82.4)	3	(17.6)	0.010 *	8	(47.1)	9	(52.9)	0.574
Higher	24	(51.1)	23	(48.9)		21	(44.7)	26	(55.3)		18	(38.3)	29	(61.7)	
**Social isolation**															
Not isolated	14	(38.9)	22	(61.1)	0.181	15	(41.7)	21	(58.3)	0.093	12	(33.3)	24	(66.7)	0.035 *
Isolated	7	(63.6)	4	(36.4)		8	(72.7)	3	(27.3)		8	(72.7)	3	(27.3)	
**Household decision making (purchases for daily needs)**															
By myself	16	(53.3)	14	(46.7)	0.898	16	(53.3)	14	(46.7)	0.611	14	(46.7)	16	(53.3)	0.255
Spouse/partner	2	(66.7)	1	(33.3)		2	(66.7)	1	(33.3)		0	(0.0)	3	(100.0)	
Spouse/partner jointly	9	(64.3)	5	(35.7)		9	(64.3)	5	(35.7)		7	(50.0)	7	(50.0)	
Someone else	10	(52.6)	9	(47.4)		8	(42.1)	11	(57.9)		5	(26.3)	14	(73.7)	

* *p* < 0.05

**Table 6:** Factors associated with behaviors toward NCDs prevention among middle-aged and older women in Indonesia.

Factors	Take a Walk					Blood Pressure					Blood Sugar Testing				
	No		Yes		*p*-Value	No		Yes		*p*-Value	No		Yes		*p*-Value
**Age**															
45–59 years old	19	(82.6)	4	(17.4)	0.016 *	2	(8.3)	22	(91.7)	0.548	10	(43.5)	13	(56.5)	0.792
60 years and older	22	(51.2)	21	(48.8)		1	(2.4)	41	(97.6)		16	(37.2)	27	(62.8)	
**Duration of residence**															
10–19 years	5	(83.3)	1	(16.7)	0.266	0	(0.0)	6	(100.0)	1.000	3	(50.0)	3	(50.0)	0.493
20–29 years	7	(77.8)	2	(22.2)		0	(0.0)	9	(100.0)		2	(22.2)	7	(77.8)	
30 years and above	26	(55.3)	21	(44.7)		3	(6.4)	44	(93.6)		18	(37.5)	30	(62.5)	
**Living alone**															
No	36	(61.0)	23	(39.0)	0.701	3	(5.1)	56	(94.9)	1.000	21	(35.6)	38	(64.4)	0.102
Yes	5	(71.4)	2	(28.6)		0	(0.0)	7	(100.0)		5	(71.4)	2	(28.6)	
**Satisfaction with income**															
Unsatisfied	9	(81.8)	2	(18.2)	0.178	0	(0.0)	11	(100.0)	1.000	4	(36.4)	7	(63.6)	1.000
Satisfied	27	(57.4)	20	(42.6)		2	(4.3)	45	(95.7)		20	(41.7)	28	(58.3)	
**Social participation**															
Lower	12	(70.6)	5	(29.4)	0.397	0	(0.0)	16	(100.0)	0.170	3	(18.8)	13	(81.3)	0.780
Higher	27	(57.4)	20	(42.6)		1	(5.3)	18	(94.7)		10	(52.6)	9	(47.4)	
**Social isolation**															
Not isolated	19	(52.8)	17	(47.2)	0.159	0	(0.0)	27	(100.0)	0.005	13	(48.1)	14	(51.9)	0.055
Isolated	9	(81.8)	2	(18.2)		2	(50.0)	2	(50.0)		0	(0.0)	4	(10.0)	
**Household decision making (purchases for daily needs)**															
By myself	18	(60.0)	12	(40.0)	0.432	0	(0.0)	25	(100.0)	0.002*	13	(50.0)	13	(50.0)	0.054
Spouse/partner	3	(100.0)	0	(0.0)		2	(50.0)	2	(50.0)		0	(0.0)	4	(100.0)	
Spouse/partner jointly	10	(71.4)	4	(28.6)		0	(0.0)	20	(100.0)		4	(21.1)	15	(78.9)	
Someone else	10	(52.6)	9	(47.4)		1	(5.9)	16	(94.1)		9	(52.9)	8	(47.1)	

* *p* < 0.05.

**Table 7:** Results of multiple regression analysis of physical health status among middle-aged and older women in Indonesia.

Factors	BMI		SBP		DBP		Handgrip Strength		Gait Speed	
	β	*p*-Value	β	*p*-Value	β	*p*-Value	β	*p*-Value	β	*p*-Value
**Age**	−0.03	0.639	1.69	<0.001 *	0.58	0.002 *	−0.16	0.020 *	−0.01	0.003 *
**Eating behavior**										
Consume a well-balanced diet	0.99	0.540	8.94	0.259	5.40	0.225	3.91	0.018 *	0.160	0.068
Eat breakfast daily	−1.10	0.490	−6.33	0.425	3.61	0.419	−2.17	0.169	−0.153	0.093
Avoid foods with high sugar	1.38	0.365	3.05	0.686	0.670	0.869	1.07	0.502	−0.004	0.949
content	−1.96	0.327	12.00	0.221	−0.31	0.955	−1.18	0.567	0.09	0.400
Avoid fatty foods	1.52	0.318	−16.86	0.028 *	−0.75	0.858	0.92	0.530	0.02	0.756
**Physical activity**										
Regular exercise	0.05	0.978	−3.12	0.681	−1.15	0.787	−0.85	0.598	−0.022	0.793
Take a walk	−0.06	0.361	−9.20	0.248	−7.99	0.087	−1.43	0.403	0.011	0.905
**Sleep**										
Sleep well	0.56	0.737	−12.97	0.118	−2.02	0.650	−0.40	0.815	−0.076	0.421
**Health checkups**										
Blood pressure	−2.64	0.361	−21.52	0.132	−5.46	0.493	1.95	0.486	0.241	0.091
Blood sugar testing	−1.13	0.496	−12.67	0.123	−6.96	0.131	−2.39	0.144	−0.087	0.328
Blood cholesterol testing	0.004	0.998	10.48	0.183	3.43	0.436	4.26	0.009 *	0.087	0.296

* *p* < 0.05.
